# Dual Deletion of the Sirtuins SIRT2 and SIRT3 Impacts on Metabolism and Inflammatory Responses of Macrophages and Protects From Endotoxemia

**DOI:** 10.3389/fimmu.2019.02713

**Published:** 2019-11-26

**Authors:** Tytti Heinonen, Eleonora Ciarlo, Ersilia Rigoni, Jean Regina, Didier Le Roy, Thierry Roger

**Affiliations:** Infectious Diseases Service, Department of Medicine, Lausanne University Hospital and University of Lausanne, Lausanne, Switzerland

**Keywords:** sirtuins, innate immunity, macrophage, sepsis, cytokine, inflammation, metabolism, endotoxemia

## Abstract

Sirtuin 2 (SIRT2) and SIRT3 are cytoplasmic and mitochondrial NAD-dependent deacetylases. SIRT2 and SIRT3 target proteins involved in metabolic, proliferation and inflammation pathways and have been implicated in the pathogenesis of neurodegenerative, metabolic and oncologic disorders. Both pro- and anti-inflammatory effects have been attributed to SIRT2 and SIRT3, and single deficiency in SIRT2 or SIRT3 had minor or no impact on antimicrobial innate immune responses. Here, we generated a SIRT2/3 double deficient mouse line to study the interactions between SIRT2 and SIRT3. SIRT2/3^−/−^ mice developed normally and showed subtle alterations of immune cell populations in the bone marrow, thymus, spleen, blood and peritoneal cavity that contained notably more anti-inflammatory B-1a cells and less NK cells. *In vitro*, SIRT2/3^−/−^ macrophages favored fatty acid oxidation (FAO) over glycolysis and produced increased levels of both proinflammatory and anti-inflammatory cytokines. In line with metabolic adaptation and increased numbers of peritoneal B-1a cells, SIRT2/3^−/−^ mice were robustly protected from endotoxemia. Yet, SIRT2/3 double deficiency did not modify endotoxin tolerance. Overall, these data suggest that sirtuins can act in concert or compensate each other for certain immune functions, a parameter to be considered for drug development. Moreover, inhibitors targeting multiple sirtuins developed for clinical purposes may be useful to treat inflammatory diseases.

## Introduction

Sentinel immune cells like monocytes/macrophages, dendritic cells (DCs) and polymorphonuclear neutrophils (PMNs) sense microbial- and danger-associated signals through pattern recognition receptors (PRRs) expressed at cell surface, in endosomes and in cytoplasm. The main families of PRRs are Toll-like receptors (TLRs), C-type lectin receptors, NOD-like receptors, RIG-I-like receptors and cytosolic DNA sensors (1, 2). Triggering PRRs through microbial and endogenous agonists activates mitogen-activated protein kinase (MAPK), NF-κB and interferon-related factor signal transduction pathways. Signaling leads to the production of effector molecules, such as cytokines, critical to activate innate and adaptive immunity. Tight regulation of inflammation and innate immune responses is vital for controlling microbial invasion while ensuring prompt tissue repair and return to homeostasis. Hence, innovative immunomodulatory therapies have been proposed to fight severe infections and sepsis (3–8).

Mammals express seven sirtuins (SIRT1-7). Sirtuins are NAD^+^-dependent enzymes, originally described as histone deacetylases (HDACs). Each sirtuin potentially targets thousands of non-histone proteins (9). SIRT1, SIRT6, and SIRT7 localize mainly in the nucleus, SIRT2 in the cytoplasm, and SIRT3-5 in the mitochondria. Shuttling between organelles have been observed for several sirtuins, for example SIRT2 and SIRT3 into the nucleus and SIRT5 into the cytoplasm (10). Besides a deacetylase activity described for all but SIRT4, sirtuins function as decrotonylase (SIRT1-3), demyristylase (SIRT2), ADP-ribosyltransferase (SIRT4, SIRT6), lipoamidase (SIRT4), demalonylase, deglutarylase, and desuccinylase (SIRT5), and deacylase (SIRT6) (11).

SIRT2 is the most expressed sirtuin in the brain and myeloid cells (12, 13). SIRT2 regulates cellular stability and division by targeting tubulin in the cytoplasm (14) and acting as a mitotic checkpoint in the nucleus during the G2/M phase transition (15). SIRT2 is involved in myelogenesis and other brain functions and is a promising target for treating neurodegenerative conditions among which Parkinson's disease and Huntington's disease (16). SIRT2 is a tumor suppressor gene but has also been associated with tumorigenesis (17). SIRT2 is likely linked to disease progression through cell metabolism regulation. SIRT2 inhibits glycolysis and adipogenesis and promotes lipolysis, gluconeogenesis and the pentose phosphate pathway (PPP), possibly in a cell and disease dependent manner (13, 18–22). Accordingly, SIRT2 plays a role in obesity, type 2 diabetes and other metabolic disorders (18). The picture is not clear regarding inflammatory processes. SIRT2 deficiency has been reported to stimulate, inhibit and have no effect on the activation NF-κB p65 and MAPKs, the expression of cytokines and the development of inflammatory and autoimmune diseases (13, 23–32).

SIRT3 is expressed ubiquitously and its expression increases upon caloric restriction and other stress conditions. SIRT3 is the main mitochondrial deacetylase and a major regulator of cell metabolism. SIRT3 promotes tricarboxylic acid cycle and electron transport chain, ketogenesis, fatty acid oxidation, brown adipose tissue thermogenesis and urea cycle (33–37). SIRT3 inhibits oxidative stress by activating isocitrate dehydrogenase 2 (IDH2) and superoxide dismutase 2 (SOD2) (38–40). Through the regulation of metabolism and redox homeostasis, SIRT3 protects from age-associated metabolic, cardiovascular and neurodegenerative diseases. SIRT3 also counteracts the development of inflammation-related disorders, although some studies suggest that SIRT3 does not impact on inflammatory responses (41–46). Finally, SIRT3 was shown to drive both pro-tumorigenic and tumor-suppressive effects (47).

HDACs may act in concert or compensate each other as suggested by the role played by SIRT1, HDAC5, HDAC6, and HDAC9 in dampening regulatory T cells (Tregs) and by SIRT1, SIRT3, and SIRT6 in regulating metabolic adaptation to inflammation (48–51). As a consequence, deficiency in several sirtuins might amplify or reveal phenotypes undetectable in single knockouts. Indeed, dual deletion of SIRT3 and SIRT5 showed some impact on antimicrobial host defense mechanisms not seen in - SIRT3 and SIRT5 single deficient mice (43, 52, 53). SIRT2 and SIRT3 do not seem to share targets, but they both impact on ROS detoxification at different levels. SIRT2 regulates through FOXO1 the transcription of genes encoding for ROS detoxifying enzymes while SIRT3 regulates the activity of the enzymes (38–40). Thus, the absence of SIRT2 or SIRT3 could be compensated by an increased activity of the other sirtuin. To challenge our assumption, we generated a SIRT2/3 double deficient mouse line. We show that the double deletion of SIRT2 and SIRT3 impacts on some metabolic and immune parameters not observed in single knockouts. Importantly from a translational perspective, SIRT2/3 deficient mice were protected from endotoxemia. This information is valuable considering that inhibitors targeting multiple sirtuins are developed for clinical purposes.

## Materials and Methods

### Ethics Statement

Animal experiments were approved by the Service des Affaires Vétérinaires, Direction Générale de l'Agriculture, de la Viticulture et des Affaires Vétérinaires (DGAV), état de Vaud (Epalinges, Switzerland) under authorizations 876.9 and 877.9 and performed according to Swiss and ARRIVE guidelines.

### Mice

SIRT2^−/−^ and SIRT3^−/−^ C57BL/6J mice have been described (54, 55). SIRT3^−/−^ females were crossed with SIRT2^−/−^ males to obtain 53 female and 47 male SIRT2/3^+/−^ mice. SIRT2/3^+/−^ mice were crossed to obtain 312 F2 mice. We identified 7 female and 3 male double knockout mice that were used to establish the SIRT2/3^−/−^ mouse line. For genotyping purposes, DNA was extracted and analyzed by PCR using the Mouse Direct PCR Kit (Bimake, Houston, TX), primers pairs (Supplementary Information) and a QuantStudio™ 12K Flex system (Life Technologies, Carlsbad, CA). Mice used in this study were 7–14-week old, housed under specific pathogen-free conditions and exempt of mouse hepatitis virus and murine norovirus.

### Cells and Reagents

Bone marrow (BM) cells were cultured for 7 days in RPMI 1640 (Life Technologies, Carlsbad, CA) supplemented with 1% penicillin-streptomycin (Invitrogen, Carlsbad CA), 10% heat inactivated fetal bovine serum (FBS; Biochrom GmbH, Berlin, Germany) and 50 U/ml macrophage colony-stimulating factor (M-CSF; ImmunoTools, Friesoythe, Germany) to obtain BM derived macrophages (BMDMs) (56). BMDMs were seeded in 96-well or 24-well plates (2 × 10^5^ or 1 × 10^6^ cells/well) without M-CSF for stimulation and phagocytosis/killing experiments, respectively. Peritoneal cells were obtained through a peritoneal lavage performed using 5 ml ice-cold RPMI (57). Cells were enumerated and seeded in 96-well plates (2 × 10^5^ cells/well) in RPMI containing 1% penicillin-streptomycin and 10% FBS. BMDMs and peritoneal cells were stimulated with ultrapure LPS from *Salmonella minnesota* (List Biological Laboratories, Campbell, CA), Pam_3_CSK_4_ (EMC microcollections, Tübingen, Germany), and CpG A 1585 (Microsynth, Balgach, Switzerland).

### RNA Analyses

RNA was extracted with the RNeasy kit (Qiagen, Hilden, Germany) and reverse transcribed with the QuantiTect reverse transcription kit (Qiagen). Real-time PCR was performed using primers (Supplementary Information) and KAPA SYBR Green Fast (Kapa Biosystems, Wilmington, MA) as described (58). Reactions were run on a QuantStudio™ 12K Flex system (Life Technologies). Gene expression was normalized to actin expression.

### Western Blot Analyses

Proteins were extracted, submitted to PAGE and transferred to nitrocellulose membranes as described (59). Membranes were incubated with primary antibodies against β-actin (4967, Cell Signaling, Danvers, MA), NF-κB p65 (8242, Cell Signaling) p44/42 MAPK (ERK1/2; 9102, Cell Signaling), phospho-p44/42 (ERK1/2; 9101, Cell Signaling), p38 MAPK (9102, Cell Signaling), phosho-p38 MAPK (9211, Cell Signaling), SIRT2 (ab67299; Abcam, Cambridge, United Kingdom), SIRT3 (5490; Cell Signaling), α-tubulin (T5168; Sigma-Aldrich, Darmstadt, Germany), and HRP-coupled secondary antibodies (31430 and 31460; Invitrogen). Blots were revealed with the enhanced chemiluminescence Western blotting system (Advansta, San Jose, CA). Images were recorded with the Fusion Fx system (Viber Lourmat, Collégien, France). Full length blots are presented in Supplementary Figure 1.

### Flow Cytometry

Single cell suspensions were incubated with Fc blocker, stained with antibodies listed in Supplementary Information and fixed with 2% paraformaldehyde. Data were acquired with an Attune Nxt flow cytometer (ThermoFisher, Waltham, MA) and analyzed using FlowJo 10.2 (FlowJo LLC, Ashland, OR). Gating strategies are presented in Supplementary Figure 2 and in Heinonen et al. (52).

### Metabolic Activity

Four × 10^4^ BMDMs per well were plated in Seahorse XFe96 plates. Glycolytic activity, mitochondrial respiration and mitochondrial flexibility were analyzed using Seahorse Glycolysis Stress and Mito Fuel Flex Test kits (Agilent, Santa Clara, CA) as recommended by the manufacturer. Two × 10^4^ BMDMs were plated in 96-well plates and grown in RPMI (Sigma-Aldrich) with 5 mM glucose. Glucose and lactate were measured with the Glucose-Glo and Lactate-Glo kits (J6021 and J5021, Promega, Madison, WI) and luminescence was recorded with a Synergy plate reader (BioTek, Winooski, VT).

### Cytokine Measurements

Cytokines and chemokines were measured in cell supernatant and plasma by ELISA (IL-6 and TNF: R&D systems, Minneapolis, MN; IL-10: Mabtech, Nacka Strand, Sweden) or by Luminex (Mouse Custom ProcartaPlex 17-plex: ENA-78/CXCL5, G-CSF, IFNγ, IL-1α, IL-1β, IL-3, IL-6, IL-10, IL-12p40, IL-17A, IL-18, IP-10/CXCL10, KC/CXCL1, MCP-1/CCL2, MIP-1α/CCL3, MIP-2/CXCL2, TNF) (Invitrogen) and recorded with a bioplex 200 (Bio-Rad, Hercules, CA) (60).

### Phagocytosis and Killing Assay

*E. coli* O18 (*E. coli*) was grown in brain heart infusion broth (Oxoid Limited, Hampshire, United Kingdom) and washed in 0.9% NaCl (61). BMDMs were incubated with Fluoresbrite^®^ Yellow Green Microspheres (Polysciences Inc., Warrington, PA, USA) or live *E. coli* at a bead or bacteria-to-cell ratio of 10:1 for 1 h to quantify phagocytosis and for 6 h to quantify killing as described (62).

### *In vivo* Models

SIRT2/3^+/+^ and SIRT2/3^−/−^ male mice (*n* = 8–10 per group) were challenged intraperitoneally with 10 mg/kg LPS from *E. coli* O111:B4. Blood was collected 0, 1, 3, and 7 days post-challenge to quantify cytokines by Luminex, analyze cell populations by flow cytometry, and perform whole blood assays as described (63). Body weight loss, severity score and survival were monitored at least twice daily. The severity score was graded from 0 to 6 based on animal motility and aspect. Two to three operators performed animal follow-up (64).

### Statistical Analyses

Graphics represent data obtained from individual mice (dots), or box with min to max whiskers. Data from different groups were analyzed for normal distribution and homogeneity of variances and compared with the appropriate parametric (two-tailed unpaired Student's *t*-test) or non-parametric (two-tailed Mann-Whitney test) statistical test. For gravity score, area under the curve was used for analysis. Survival was analyzed using the Kaplan-Meier method. *P*-values were two-sided, and *P* < 0.05 was considered to indicate statistical significance. ^*^P ≤ 0.05; ^**^P ≤ 0.01; ^***^P ≤ 0.005. Analyses were performed using PRISM 8.0.1 (GraphPad Software, San Diego, CA).

## Results

### SIRT2/3^−/−^ Mice Develop Without Abnormalities

**Figure 1:**
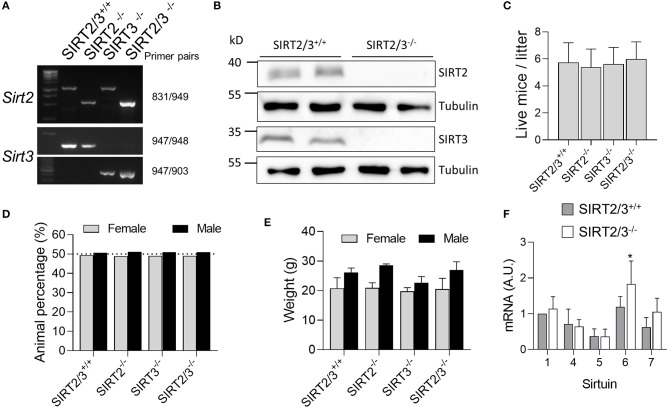
Establishment of a SIRT2/3^−/−^ mouse line. **(A)** Genotyping of SIRT2/3^+/+^, SIRT2^−/−^, SIRT3^−/−^, and SIRT2/3^−/−^ mice. Genomic DNA was amplified by PCR using the primer pairs specified on the right. Reaction mixtures were electrophoresed through agarose gels before imaging. **(B)** Western blot analysis of SIRT2, SIRT3 and tubulin expression in SIRT2/3^+/+^ and SIRT2/3^−/−^ BMDMs (Full blots are presented in Supplementary Figure 1). **(C,D)** Number of individuals **(C)** and female/male distribution **(D)** of SIRT2/3^+/+^, SIRT2^−/−^, SIRT3^−/−^ and SIRT2/3^−/−^ litters. **(E)** Weight of SIRT2/3^+/+^, SIRT2^−/−^, SIRT3^−/−^, and SIRT2/3^−/−^ adult female and male mice. **(F)** mRNA expression of SIRT1 and SIRT4-7 in SIRT2/3^−/−^ BMDMs were quantified by RT-qPCR and normalized to actin mRNA. Data are mean ± SD of eight mice analyzed in triplicate. *P < 0.05.

SIRT2^−/−^ and SIRT3^−/−^ mice were crossed to generate a F2 population (see **Materials and Methods**). Among 312 F2 mice, we identified 10 double knockouts that were used to establish the SIRT2/3^−/−^ mouse line. The truncation of the *Sirt2* and *Sirt3* genes and the absence of SIRT2 and SIRT3 protein expression were confirmed by PCR (Figure 1A) and Western blotting (Figure 1B). The size of litters (Figure 1C) and the female/male sex ratio of litters (Figure 1D) were similar in the SIRT2/3^+/+^, SIRT2^−/−^, SIRT3^−/−^, and SIR2/3^−/−^ mouse lines. The weight of adult female and male mice were comparable among mouse lines (Figure 1E). Mouse development was normal, and no macroscopic abnormalities were detected upon autopsy. The expression levels of Sirt1, 4, 5, and 7 mRNA were not affected while the expression of Sirt6 mRNA was increased 1.5-fold in SIRT2/3^−/−^ BMDMs (Figure 1F).

### SIRT2/3^−/−^ Mice Have Minor Alterations of Leukocyte Subpopulations

SIRT2 and SIRT3 are expressed by all major immune cell subpopulations (13, 43) but their role in the development of myeloid cells is unknown. We quantified leukocyte populations in primary and secondary lymphoid organs, blood and peritoneum of SIRT2/3^+/+^, SIRT2^−/−^, SIRT3^−/−^, and SIRT2/3^−/−^ mice. In the bone marrow, the number of CD45^+^ cells was similar in all mouse lines (Table 1). The frequency of T cells, conventional DCs (cDCs), plasmacytoid DCs (pDCs) and granulocytes was not affected in knockout mouse lines. The proportion of Ly6C^low^ (alternative) monocytes was slightly reduced (<10%) in SIRT2/3^−/−^ mice, as it was in SIRT2^−/−^ and SIRT3^−/−^ mice. In contrast, the frequency of B cells was 30% higher in SIRT2/3^−/−^ mice. In the thymus, SIRT2/3^−/−^ mice expressed normal proportions and absolute numbers of CD4/CD8 double negative (DN) thymocytes, DN1-DN4 thymocytes, and CD4 and CD8 single positive thymocytes and 4% less CD4^+^ CD8^+^ double positive thymocytes (Table 2). The spleen of SIRT2/3^+/+^, SIRT2^−/−^, SIRT3^−/−^, and SIRT2/3^−/−^ mice contained comparable populations of CD4^+^ and CD8^+^ T cells and monocytes (Table 3). However, the spleen of SIRT2/3^−/−^ mice contained 19% more naïve CD4^+^ T cells and 11% more B cells, as seen in the bone marrow, but 17% less CD11c^+^ DCs and 39% less Ly6G^+^ granulocytes (Table 3). Taken all together, SIRT2/3 deficiency had no dramatic impact on leukocyte development.

**Table 1:** Bone marrow cell subsets.

	**SIRT2/3^**+/+**^ (*n* = 4)**	**SIRT2^**−/−**^ (*n* = 4)**	**SIRT3^**−/−**^ (*n* = 4)**	**SIRT2/3^**−/−**^ (*n* = 4)**
B220^+^ B cells	13.2 ± 1.9	15.5 ± 3.8	17.2 ± 0.7	17.4 ± 1.3
CD3^+^ T cells	4.6 ± 0.9	3.5 ± 1.1	5.2 ± 1.1	3.6 ± 1.1
CD11c^+^ DCs	3.8 ± 0.3	3.3 ± 0.2	3.8 ± 0.3	3.6 ± 0.2
pCDs	63.6 ± 2.2	64.3 ± 2.6	62.2 ± 0.9	67.4 ± 4.0
cDCs	33.4 ± 2.0	32.8 ± 2.7	34.2 ± 0.9	29.9 ± 3.7
cDC1 (CD11b^−^)	51.0 ± 2.9	49.7 ± 8.2	48.0 ± 4.6	52.7 ± 3.6
cDC2 (CD11b^+^)	45.8 ± 2.9	47.4 ± 8.5	48.0 ± 4.6	44.3 ± 3.4
Ly6G^+^ Ly6C^−^ granulocytes	48.1 ± 3.3	48.2 ± 4.2	44.3 ± 0.9	44.9 ± 1.0
Ly6G^−^ Ly6C^+^ monocytes	15.4 ± 0.8	15.3 ± 2.4	15.0 ± 0.7	15.3 ± 0.7
Ly6C^low^ alternative monocytes	44.9 ± 0.9	40.4 ± 2.0	38.9 ± 2.2	40.7 ± 2.8
Ly6C^high^ classical monocytes	48.0 ± 0.5	51.8 ± 1.7	52.2 ± 1.8	50.1 ± 2.9

*Data are mean ± SD of four mice per group and are expressed as the percentage of CD45^+^ cells (B220^+^, CD3^+^, CD11c^+^, Ly6G^+^Ly6C^−^, Ly6G^−^Ly6C^+^) or the percentage of parental cells. The number of CD45^+^ cells per leg was 14.4 ± 2.5, 14.9 ± 2.3, 12.0 ± 3.1, and 12.6 ± 4.2 × 10^6^ cells for SIRT2/3^+/+^, SIRT2^−/−^, SIRT3^−/−^, and SIRT2/3^−/−^ mice. Gray background: P < 0.05 vs. SIRT2/3^+/+^ mice*.

**Table 2:** Thymic cell subsets.

	**SIRT2/3^**+/+**^ (*n* = 4)**	**SIRT2^**−/−**^ (*n* = 4)**	**SIRT3^**−/−**^ (*n* = 4)**	**SIRT2/3^**−/−**^ (*n* = 4)**
CD4^−^ CD8^−^	3.3 ± 0.2	3.3 ± 0.3	3.9 ± 0.6	4.1 ± 0.4
DN1 (CD25^−^ CD44^+^)	20.2 ± 3.2	21.1 ± 0.6	21.9 ± 6.8	21.5 ± 1.7
DN2 (CD25^+^ CD44^+^)	21.0 ± 2.4	19.8 ± 2.3	20.9 ± 1.7	21.2 ± 2.1
DN3 (CD25^+^ CD44^−^)	35.7 ± 2.5	35.3 ± 1.2	32.6 ± 2.0	32.0 ± 1.1
DN4 (CD25^−^ CD44^−^)	23.1 ± 1.5	23.8 ± 2.7	24.6 ± 3.6	25.3 ± 1.9
CD4^+^ CD8^+^	84.6 ± 1.2	82.4 ± 0.7	80.9 ± 3.1	81.1 ± 0.2
CD4^+^	6.8 ± 0.7	8.1 ± 0.4	8.7 ± 1.9	8.2 ± 0.5
CD8^+^	1.6 ± 0.3	2.3 ± 0.2	2.1 ± 0.7	2.0 ± 0.4

*Data are mean ± SD of four mice per group expressed as percentage of total thymocytes (CD4^−^ CD8^−^, CD4^+^ CD8^+^, CD4^+^, CD8^+^) or the percentage of CD4^−^ CD8^−^ cells (CD25^−^ CD44^+^, CD25^+^ CD44^+^, CD25^+^ CD44^−^, CD25^−^ CD44^−^). Thymus weights were 71.3 ± 8.6, 107.0 ± 20.9, 90.5 ± 15.6, and 89.5 ± 5.3 mg for SIRT2/3^+/+^, SIRT2^−/−^, SIRT3^−/−^, and SIRT2/3^−/−^ mice. Gray background: P < 0.05 vs. SIRT2/3^+/+^ mice*.

**Table 3:** Splenic cell subsets.

	**SIRT2/3^**+/+**^ (*n* = 4)**	**SIRT2^**−/−**^ (*n* = 4)**	**SIRT3^**−/−**^ (*n* = 4)**	**SIRT2/3^**−/−**^ (*n* = 4)**
B220^+^ B cells	47.1 ± 0.8	48.9 ± 1.3	47.1 ± 2.5	52.2 ± 2.2
Mature (CD23^+^ IgD^+^)	70.0 ± 1.8	67.2 ± 1.6	66.8 ± 2.4	67.3 ± 2.4
Immature (non-CD23^+^IgD^+^)	30.0 ± 1.8	32.8 ± 1.6	33.2 ± 2.4	32.7 ± 2.4
CD3^+^ T cells (%)	35.6 ± 1.6	36.3 ± 1.9	37.9 ± 2.7	34.0 ± 1.6
CD4^+^	56.0 ± 2.0	58.4 ± 1.6	58.1 ± 0.8	59.8 ± 3.0
Naive	50.0 ± 2.0	61.4 ± 4.1	56.6 ± 5.3	59.3 ± 4.5
Memory	18.4 ± 2.2	13.6 ± 1.3	13.7 ± 1.8	15.2 ± 1.4
CD8^+^	38.6 ± 1.7	36.6 ± 2.0	37.0 ± 0.7	35.0 ± 3.3
Naive	53.0 ± 3.7	58.7 ± 4.7	55.3 ± 4.4	61.2 ± 5.5
Memory	9.5 ± 1.2	7.9 ± 1.5	9.0 ± 1.6	8.4 ± 2.3
CD4^−^ CD8^−^	5.0 ± 0.4	4.7 ± 0.5	4.7 ± 0.2	5.0 ± 0.8
CD11c^+^ DCs	4.2 ± 0.3	3.9 ± 0.2	4.2 ± 0.7	3.5 ± 0.3
pDCs	22.1 ± 2.3	23.4 ± 2.5	19.8 ± 1.6	24.3 ± 3.1
cDC	77.6 ± 2.3	76.2 ± 2.5	79.8 ± 1.6	75.2 ± 3.0
cDC1 (CD11b^−^)	41.1 ± 8.9	34.4 ± 1.3	32.3 ± 0.9	35.5 ± 3.0
cDC2 (CD11b^+^)	40.5 ± 5.4	39.9 ± 2.9	45.8 ± 1.3	38.2 ± 2.6
Ly6G^+^ Ly6C^−^ granulocytes	2.8 ± 0.7	2.5 ± 0.9	1.3 ± 0.1	1.7 ± 0.3
Ly6G^−^ Ly6C^+^ monocytes	2.4 ± 0.6	2.8 ± 0.1	3.3 ± 0.2	2.7 ± 0.4
Ly6C^low^ alternative monocytes	41.7 ± 7.1	30.9 ± 9.3	48.0 ± 2.1	35.3 ± 6.5
Ly6C^high^ classical monocytes	21.8 ± 7.9	31.2 ± 4.3	13.8 ± 2.6	21.5 ± 3.9

*Data are mean ± SD of four mice per group and expressed as percentage of total cells (B220^+^, CD3^+^, CD11c^+^, Ly6G^+^ Ly6C^−^, Ly6G^−^ Ly6C^+^) or percentage of parental cells. Spleen weights were 103.8 ± 10.3, 119.3 ± 20.0, 94.5 ± 7.5, and 108.8 ± 10.0 mg for SIRT2/3^+/+^, SIRT2^−/−^, SIRT3^−/−^, and SIRT2/3^−/−^ mice. Gray background: P < 0.05 vs. SIRT2/3^+/+^ mice*.

**Figure 2:**
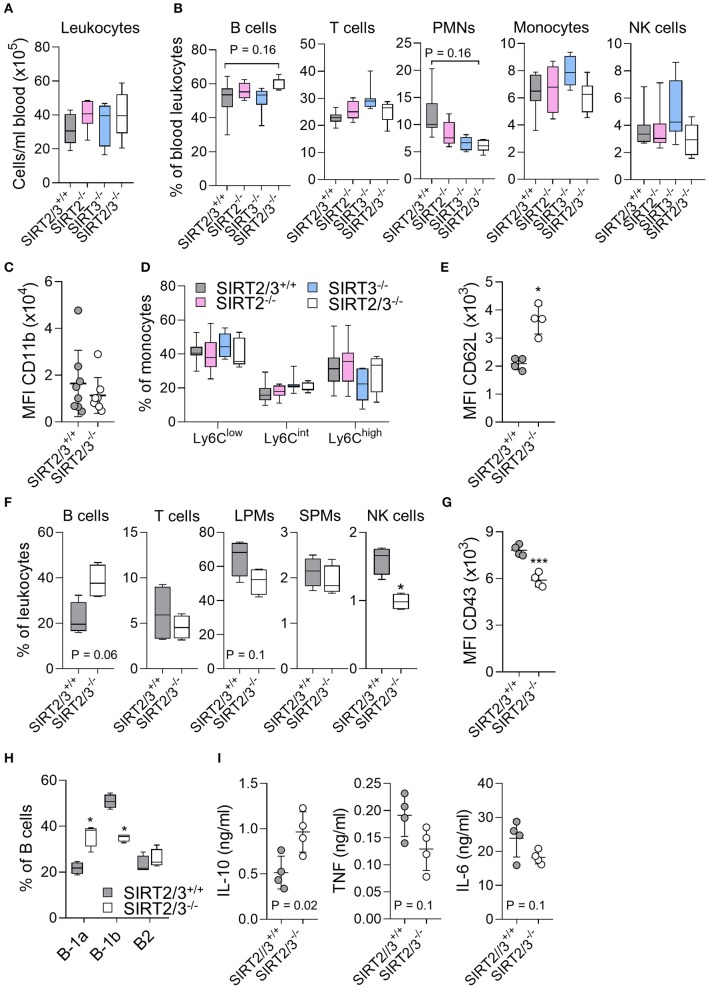
SIRT2/3^−/−^ mice have minor alterations of blood and peritoneal leukocyte subpopulations. **(A–E)** Absolute number of CD45^+^ leukocytes **(A)** and percentages of CD19^+^ B cells, CD3^+^ T cells, Ly6G^+^ PMNs, CD11b^+^Ly6G^−^ monocytes and NK1.1^+^ NK cells **(B)** in peripheral blood of SIRT2/3^+/+^, SIRT2^−/−^, SIRT3^−/−^, and SIRT2/3^−/−^ mice. **(C)** Mean fluorescence intensity (MFI) of CD11b expressed by SIRT2/3^+/+^ and SIRT2/3^−/−^ PMNs. **(D)** Percentage of Ly6C low, intermediate and high expressing monocytes. **(E)** MFI of CD62L expressed by blood SIRT2/3^+/+^ and SIRT2/3^−/−^ NK cells. **(F)** Percentage of B cells, T cells, large peritoneal macrophages (LPMs), small peritoneal macrophages (SPMs) and NK cells in the peritoneal cavity of SIRT2/3^+/+^ and SIRT2/3^−/−^ mice. **(G)** MFI of CD43 expressed by peritoneal SIRT2/3^+/+^ and SIRT2/3^−/−^ NK cells. **(H)** Percentage of B-1a (CD23^−^ CD5^+^), B-1b (CD23^−^ CD5^−^) and B-2 (CD23^+^) cells among B cells. **(I)** Peritoneal cells were exposed for 24 h to 10 ng/ml LPS. The concentrations of TNF, IL-6 and IL-10 in cell culture supernatants were measured by ELISA. Data were obtained from eight **(A–D)** or four **(E–I)** mice per group. Each dot represents one mouse. *P < 0.05; ***P < 0.005. Gating strategies are presented in Supplementary Figure 3 and in Heinonen et al. (52).

Blood leukocytes play a key role in sensing MAMPs/DAMPs and are quickly recruited to inflamed tissues. The number of leukocytes and the frequencies of B cells, T cells, PMNs, monocytes and NK cells were similar in SIRT2/3^+/+^, SIRT2^−/−^, SIRT3^−/−^, and SIRT2/3^−/−^ mice (Figures 2A,B), although we could observe an increased frequency of B cells and decreased frequency of PMNs in the blood of SIRT2/3^−/−^ mice. The egress of PMNs from the bone marrow follows a circadian rhythm, a process that is modulated by sirtuins (65). Aged neutrophils express increased levels of CD11b (66). CD11b mean fluorescence intensity (MFI) of SIRT2/3^+/+^ and SIRT2/3^−/−^ PMNs was alike (Figure 2C), suggesting that SIRT2 and SIRT3 do no impact on PMNs release from the bone marrow. The frequency of Ly6C^low^, Ly6C^int^, and Ly6C^high^ monocyte subpopulations were similar in all mouse lines (Figure 2D). Hence, the reduced number of Ly6C^low^ monocytes in the bone marrow of knockout mice (Table 1) had no perceptible impact on blood monocytes. CD62L, a homing receptor for secondary lymphoid organs, is expressed by NK cells at steady-state (67). CD62L MFI was 1.7- to 2-fold higher in NK cells from SIRT2/3^−/−^ mice (Figure 2E).

The peritoneal cavity of mice contains mainly B cells, T cells, macrophages and NK cells (68). Peritoneal macrophages are divided into large peritoneal macrophages (LPMs) and small peritoneal macrophages (SPMs). LPMs are self-renewing macrophages with homeostatic functions and represent the main macrophage population (90% of all macrophages) expressed at baseline in the peritoneum. SPMs derived from blood inflammatory monocytes quickly exceed LPMs upon infection or inflammation (69). The peritoneal cavity of SIRT2/3^−/−^ mice contained 1.7-fold less NK cells, while the other leukocyte subpopulations were not statistically significantly different (Figure 2F). Beside a reduced number of NK cells, the MFI of the activation marker CD43 expressed by NK cells was 1.3-fold lower in SIRT2/3^−/−^ mice (Figure 2G). Similar to what was observed in the bone marrow and spleen, there was 1.8-fold more B cells in the peritoneum of SIRT2/3^−/−^ mice (Figure 2F). B cells are divided into B1 (B-1a and B-1b) cells producing natural antibodies and B2 cells (70). Interestingly, the peritoneal cavity of SIRT2/3^−/−^ mice contained proportionally more B-1a cells and less B-1b cells than the one of SIRT2/3^+/+^ mice (Figure 2H). This resulted in 3.3-fold more B-1a cells, 1.4-fold more B-1b cells and subnormal number of B2 cells in SIRT2/3^−/−^ mice (SIRT2/3^+/+^ vs. SIRT2/3^−/−^: 0.9 ± 0.2 vs. 3.0 ± 0.5 × 10^5^ B-1a cells, 2.1 ± 0.8 vs. 3.0 ± 0.6 × 10^5^ B-1b cells, 0.8 ± 0.4 vs. 1.9 ± 1.2 × 10^5^ B-2 cells, *P* = 0.007, 0.05, and 0.08). To substantiate the relevance of this observation, we quantified IL-10, TNF and IL-6 production by peritoneal cells stimulated *ex vivo* for 24 h with 10 ng/ml LPS (Figure 2I). SIRT2/3^−/−^ peritoneal cells produced more IL-10 and less TNF and IL-6 than SIRT2/3^+/+^ peritoneal cells (*P* < 0.02 for IL-10). These results were in line with the fact that B-1 cells, and particularly B-1a cells, are considered to produce high levels of IL-10 at baseline and upon microbial stimulation (70–72). The proportion of LPMs was slightly reduced in the peritoneal cavity of SIRT2/3^−/−^ mice (Figure 2F). Overall, SIRT2/3 deficiency resulted in minor organ-specific alterations of the main leukocyte subpopulations, apart from an increased, functionally relevant, number of B-1a cells in the peritoneum.

### SIRT2/3 Deficiency Enhances Cytokine Secretion, Phagocytosis, and Killing by Macrophages

**Figure 3:**
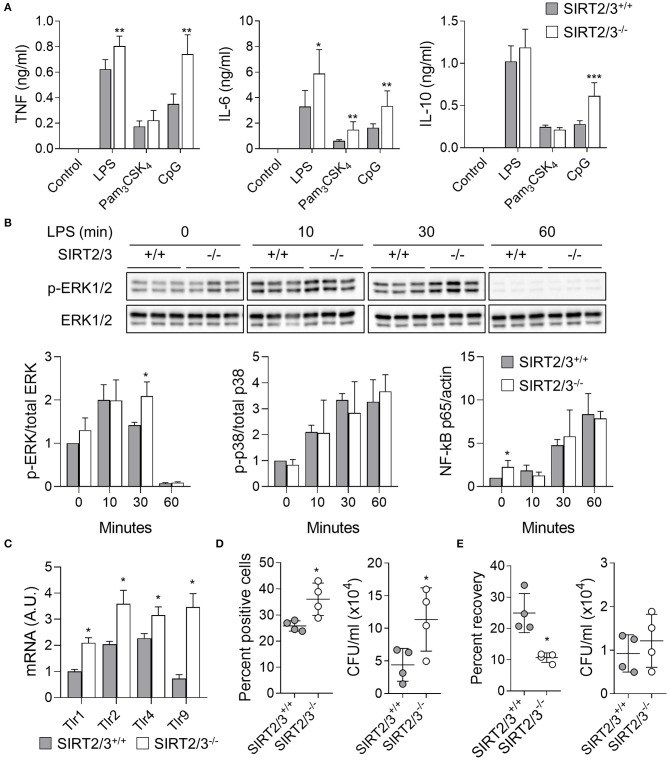
SIRT2/3 deficiency increases cytokine secretion, phagocytosis and killing by macrophages. **(A)** BMDMs were exposed to LPS (10 ng/ml), Pam_3_CSK_4_ (100 ng/ml), and CpG (1 μg/ml) for 24 h. The concentrations of TNF, IL-6, and IL-10 in cell culture supernatants were measured by ELISA. **(B)** BMDMs were exposed for 0, 10, 30, and 60 min to 10 ng/ml LPS. Phosphorylation of ERK1/2 and p38, and nuclear translocation of NF-κB p65 were measured by Western blotting. Representative blots are presented for ERK1/2 phosphorylation (Full blots are available in Supplementary Figure 1). Quantitative data are means ± SD of three mice. **(C)** Tlr1, Tlr2, Tlr4, and Tlr9 mRNA levels in BMDMs were quantified by RT-qPCR and normalized to actin mRNA levels. Data are mean ± SD of four mice analyzed in triplicate. **(D)** Phagocytosis by BMDMs exposed for 1 h to fluorescent beads (left) and live *E. coli* (right) at a bead or bacteria-to-cell ratio of 10:1. Data were analyzed by flow cytometry and colony enumeration, respectively. **(E)** Killing of *E. coli* by BMDMs. The percentage of recovery (left) was calculated by dividing the number of bacteria recovered after 6 h by the number of bacteria recovered after 1 h. Absolute counts are presented on the right. Data are mean ± SD of four mice analyzed in triplicate. Each dot represents a mouse. *P < 0.05; **P < 0.01; ***P < 0.005.

Previous studies demonstrated that the deletion of SIRT2 and SIRT3 had no consequence on the expression of PRRs and microbial product-induced cytokine production by immune cells including BMDMs (13, 43). To test if SIRT2/3 deletion influenced cytokine production, BMDMs were exposed to LPS, Pam_3_CSK_4_, and CpG (i.e., agonists of TLR4, TLR1/2, and TLR9, respectively) for 24 h before measuring the concentrations of TNF, IL-6 and IL-10 in cell culture supernatants (Figure 3A). SIRT2/3^−/−^ BMDMs produced significantly more TNF in response to LPS and CpG, more IL-6 in response to LPS, Pam_3_CSK_4_ and CpG, and more IL-10 in response to CpG. To test whether an increased intracellular signaling was associated with increased cytokine production, we quantified by Western blotting the phosphorylation of ERK1/2 and p38 MAPKs and the nuclear translocation of NF-κB p65 in BMDMs exposed to LPS for 0, 10, 30, and 60 min (Figure 3B). SIRT2/3 double deletion resulted in higher NF-κB p65 nuclear content at baseline and increased phosphorylation of ERK1/2 after 30 min of stimulation. p38 was not differentially phosphorylated in SIRT2/3^+/+^ and SIRT2/3^−/−^ BMDMs. In line with the increased intracellular signaling and cytokine production, the expression level of TLR1, TLR2, TLR4, and TLR9 mRNA was increased in SIRT2/3^−/−^ BMDMs when compared to SIRT2/3^+/+^ BMDMs (Figure 3C).

Since SIRT2 deficiency promoted phagocytosis (13), we compared the phagocytic activity over a period of 1 h of SIRT2/3^+/+^ and SIRT2/3^−/−^ BMDMs. The analysis by flow cytometry of BMDMs incubated with fluorescent beads showed a 1.4-fold higher proportion of SIRT2/3^−/−^ BMDMs having phagocytosed beads (*P* = 0.03; Figure 3D, left panel). To better reflect infectious conditions, BMDMs were exposed to live *E. coli*, and intracellular bacteria were quantified by plating cell lysates followed by the enumeration of colonies. SIRT2/3^−/−^ BMDMs ingested 2.6-fold more *E. coli* (SIRT2/3^+/+^ vs. SIRT2/3^−/−^ BMDMs: 4.4 ± 2.5 vs. 11.3 ± 4.8 × 10^4^ *E. coli, P* = 0.03; Figure 3D, right panel). The increased phagocytic activity of SIRT2/3^−/−^ BMDMs was independent from an increased expression of the phagocytic receptors integrin αM/Itgam (CD11b), Itgβ1/Itgb1 (CD29), Itgβ2/Itgb2 (CD18), Itgα5/Itga5 (CD49e), and ItgαX/Itgax (CD11c) (Supplementary Figure 2A). Since a main function of phagocytes is to kill invading microorganisms, we tested the killing of *E. coli* by BMDMs following 6 h of incubation. SIRT2/3^−/−^ BMDMs showed around 2-fold more efficient killing capacity when compared to SIRT2/3^+/+^ BMDMs as demonstrated by a reduced percentage recovery of ingested bacteria by SIRT2/3^−/−^ BMDMs (SIRT2/3^+/+^ vs. SIRT2/3^−/−^ BMDMs: 25 ± 6 vs. 11 ± 2% recovery of ingested *E. coli, P* = 0.03; Figure 3E, left panel), and similar total numbers of bacteria in SIRT2/3^+/+^ and SIRT2/3^−/−^ BMDMs (9.3 vs. 1.2 × 10^4^ total *E. coli, P* = 0.5; Figure 3E, right panel).

### SIRT2/3 Deficiency Alters Macrophage Metabolism

**Figure 4:**
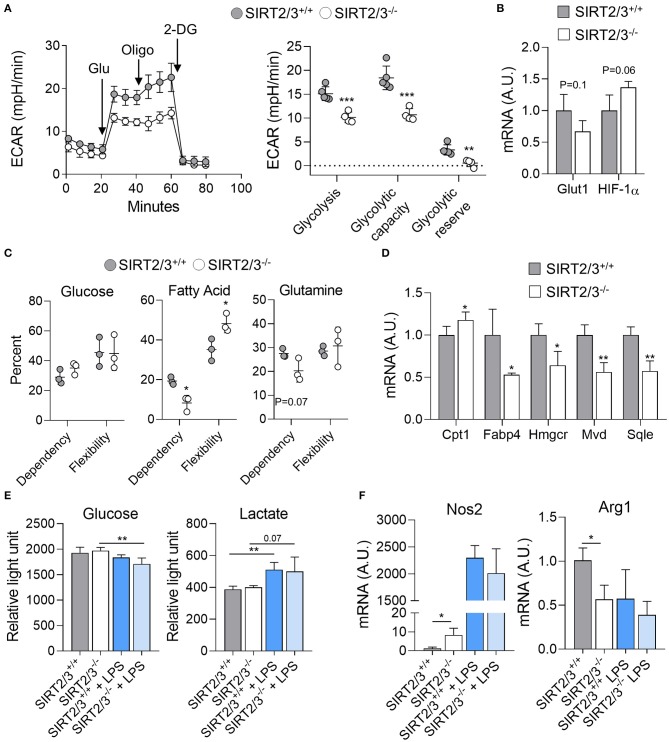
SIRT2/3 deficiency alters macrophage metabolism. **(A)** The extracellular acidification rate (ECAR) of SIRT2/3^+/+^ and SIRT2/3^−/−^ BMDMs was measured using the Seahorse technology (left) and used to calculate the glycolysis, glycolytic capacity and glycolytic reserve (right). Glu, glucose; Oligo, oligomycin; 2-DG, 2-deoxyglucose. **(B)** Glut1 and Hif1a mRNA levels in BMDMs were quantified by RT-qPCR and normalized to actin mRNA levels. **(C)** Dependency and flexibility of SIRT2/3^+/+^ and SIRT2/3^−/−^ BMDMs toward glucose, fatty acids (FA) and glutamine. **(D)** Cpt1, Fabp4, Hmgcr, Mvd and Sqle mRNA levels in BMDMs were quantified by RT-qPCR and normalized to actin mRNA levels. **(E)** Glucose consumption and lactate production by SIRT2/3^+/+^ and SIRT2/3^−/−^ BMDMs left unstimulated or stimulated for 8 h (glucose) or 24 h (lactate) with 10 ng/ml LPS. Glucose and lactate were detected using a luminescence-based assay. **(F)** Nos2 and Arg1 were quantified by RT-qPCR and normalized to actin mRNA levels. Data are means ± SD of 3–5 mice analyzed in triplicate. *P < 0.05; **P < 0.01; ***P < 0.005.

Macrophages acquire energy mainly through oxidative metabolism under steady-state and switch to glycolysis upon activation (73, 74). Since SIRT2/3 deficiency increased the cytokine response of BMDMs, though both proinflammatory and anti-inflammatory cytokines, we assumed that their glycolytic parameters were affected. The metabolic parameters of SIRT2/3^+/+^ and SIRT2/3^−/−^ BMDMs were measured using the Seahorse technology. Unexpectedly, SIRT2/3^−/−^ BMDMs displayed reduced glycolysis, glycolytic capacity and glycolytic reserve (Figure 4A). In line with this observation, SIRT2/3^−/−^ BMDMs expressed slightly reduced mRNA levels of Solute carrier family 2, member 1 also known as Glucose transporter 1 (Glut1) while the mRNA levels of Hypoxia inducible factor 1α (Hif1a) were slightly increased (1.6-fold, Figure 4B). To assess whether the lower glycolysis of SIRT2/3^−/−^ BMDMs was compensated by an increased metabolism of other sources of energy, we assessed the dependency (i.e., the necessity for a fuel to meet metabolic demand) and the flexibility (i.e., the ability to increase the usage of fuel when access to other energy sources is inhibited) of BMDMs toward glucose, fatty acids (FA) and glutamine. SIRT2/3^+/+^ and SIRT2/3^−/−^ BMDMs were equally dependent and flexible toward glucose (Figure 4C). However, SIRT2/3^−/−^ BMDMs were less dependent than SIRT2/3^+/+^ BMDMs toward FA and glutamine, but were more flexible than SIRT2/3^+/+^ BMDMs toward FA (Figure 4C). FA are metabolized in the mitochondria through FA oxidation (FAO). FAO is controlled by the rate-limiting enzyme Carnitine palmoyltransferase I (Cpt1) that facilitates FA transport to the mitochondria (74). SIRT2/3^−/−^ BMDMs expressed higher levels of Cpt1 mRNA when compared to SIRT2/3^+/+^ BMDMs, but lower levels of mRNA encoding for carrier protein and enzymes involved in cholesterol synthesis FA binding protein 4 (Fabp4), 3-Hydroxy-3-methylglutaryl-coenzyme A reductase (Hmgcr), Mevalonate decarboxylase (Mvd), and Squalene epoxidase (Sqle) (Figure 4D). Therefore, SIRT2/3 deficiency possibly favored FAO over cholesterol synthesis.

LPS stimulation induces macrophage polarization toward a classical, M1, phenotype which is linked to a metabolic shift from oxidative phosphorylation to glycolysis (73, 74). Thus, we measured the consumption of glucose and the production of lactate upon LPS stimulation. Glucose concentration in medium decreased when BMDMs were exposed for 8 h to LPS, with a slightly superior effect observed with SIRT2/3^−/−^ BMDMs (Control vs. LPS: *P* = 0.2 and 0.008 for SIRT2/3^+/+^ and SIRT2/3^−/−^ BMDMs; Figure 4E). In line with a metabolic shift, the levels of lactate increased in the medium of LPS-simulated BMDMs (Figure 4E). Overall, glucose consumption and lactate production were not different between SIRT2/3^+/+^ and SIRT2/3^−/−^ BMDMs. Although these results were unanticipated considering ECAR measurements, they could also reflect the small increase in the production of both proinflammatory and anti-inflammatory cytokines by double knockout BMDMs exposed to LPS. Finally, we questioned whether SIRT2/3^−/−^ altered macrophage polarization by measuring the mRNA expression levels of Nitric oxide synthase 2 (Nos2) and Arginase 1 (Arg1) as markers of M1 and M2 phenotypes, respectively. SIRT2/3^−/−^ macrophages expressed higher levels of Nos2 and lower levels of Arg1 at baseline, but similar levels of both mRNAs after stimulation with LPS (Figure 4F).

### SIRT2/3^−/−^ Mice Are Protected From Endotoxic Shock

**Figure 5:**
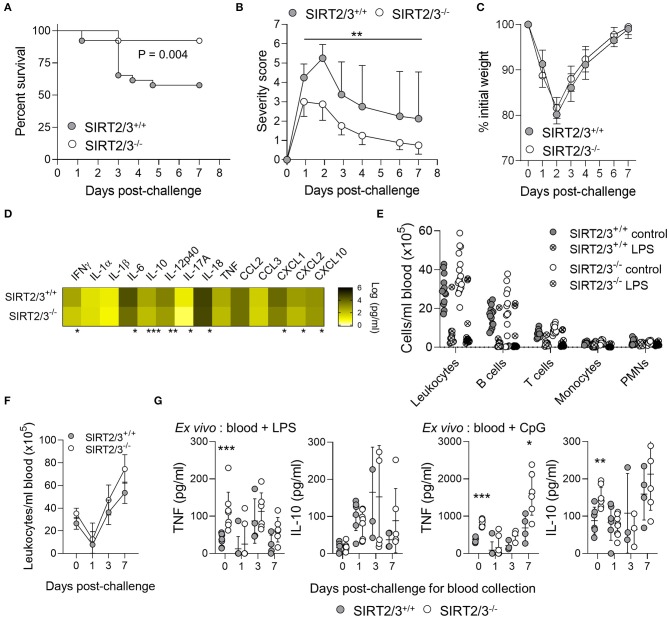
SIRT2/3^−/−^ mice are protected from endotoxic shock. Mice were challenged with 10 mg/kg LPS i.p. Survival **(A)**, severity score **(B)**, and weight loss **(C)** were recorded daily. Data are mean ± SD **(B,C)**. **(D–G)** Blood was collected 0, 1, 3, and 7 days post-challenge to quantify the concentrations of cytokines by Luminex (**D**, at day 1 post-challenge) and leukocytes by flow cytometry (**E,F**, at day 1 post-challenge in **E**). Blood collected 0, 1, 3, and 7 days post-LPS challenge was stimulated *ex vivo* for 6 h with 10 ng/ml LPS and 10 μM CpG. TNF and IL-10 concentrations were measured by ELISA **(G)**. TNF and IL-10 concentrations in blood cultured for 6 h without stimulation were below the detection limit (not depicted on the graphs). Data were obtained from 26 **(A)**, 7–8 **(B,C,F,G)**, 4 **(D)**, and 14–16 **(E)** mice per group. *P < 0.05; **P < 0.01; ***P < 0.005.

Acute inflammation induced by LPS is characterized by an early anabolic glycolytic phase followed by a catabolic adaptation phase involving FAO (50). SIRT2^−/−^ and SIRT3^−/−^ mice were shown to behave like wild-type mice in models of endotoxemia (13, 43). Yet, considering that SIRT2/3^−/−^ BMDMs stimulated with microbial products produced more proinflammatory and anti-inflammatory cytokines, and that the peritoneum of SIRT2/3^−/−^ mice contained more anti-inflammatory B-1a cells and less activated NK cells, we hypothesized that SIRT2/3 deficiency may protect mice from acute inflammation. A model of endotoxic shock was developed by challenging mice intraperitoneally with 10 mg/kg LPS. While 93% of SIRT2/3^−/−^ mice survived endotoxemia, only 58% of SIRT2/3^+/+^ mice did (*P* = 0.004; Figure 5A). In agreement, SIRT2/3^−/−^ mice showed lower severity scores than SIRT2/3^+/+^ mice (Figure 5B), but there was no noticeable differential effect on mouse weight (Figure 5C). Depletion of macrophages by clodronate liposomes injected i.p. (90% depletion, *n* = 4; *P* < 0.001) did not modify the survival profiles (*P* = 0.6), suggesting a minor impact of macrophages in our model. To assess the immune status of endotoxemic mice, blood was collected 1 day post-challenge to quantify cytokines (Figure 5D) and leukocyte subpopulations (Figure 5E). Nine out of the fourteen detectable cytokines measured by Luminex (IFNγ, IL-6, IL-10, IL-12p40, IL-17A, IL-18, KC/CXCL1, MIP-2/CXCL2, IP-10/CXCL10) were present at significantly lower concentrations in the blood of SIRT2/3^−/−^ mice. Endotoxemia was associated with a strong reduction of all blood leukocyte subpopulations (Figure 5E). Finally, we questioned whether SIRT2/3 deletion would affect endotoxin tolerance as reported for other sirtuins (24, 75–77). To address that question, blood was collected from mice challenged 0, 1, 3, and 7 days earlier with LPS. Blood was used to quantify leukocytes and to measure TNF and IL-10 response upon *ex vivo* exposure for 6 h to 10 ng/ml LPS or 10 μM CpG. At baseline, the blood of SIRT2/3^−/−^ mice contained slightly more leukocytes (Figure 5F) and produced more TNF and IL-10 in response to LPS and CpG stimulation (Figure 5G). Confirming the induction of endotoxin tolerance, TNF production induced by LPS and CpG was decreased while IL-10 production induced by LPS was enhanced when comparing the reactivity of blood from SIRT2/3^+/+^ and SIRT2/3^−/−^ mice challenged 1 day earlier with LPS with that of untouched mice (Figure 5G). The production of TNF and IL-10 returned roughly to those measured under baseline conditions using blood collected from mice challenged 1 week earlier with LPS. Overall, SIRT2/3 double deletion did not seem to affect the induction of endotoxin tolerance.

## Discussion

Sirtuins share structural and functional features and shuttle between cellular compartments. Hence they may compensate the absence of one sirtuin in single knockout mice. Here, we described a germinal SIRT2/3 double deficient mouse line. We elected to delete SIRT2 and SIRT3 because SIRT2 is the overall most expressed sirtuin and SIRT3 the predominant mitochondrial sirtuin in myeloid cells (13, 43). The new SIRT2/3^−/−^ mouse line developed normally without apparent defects. Yet, SIRT2/3 deficiency altered to some extent the frequency of immune cells in several immune compartments and the metabolism and functions of macrophages. Importantly, SIRT2/3^−/−^ mice were protected from endotoxemia, contrary to SIRT2^−/−^ and SIRT3^−/−^ mice that behaved like wild-type mice (13, 43). Altogether, these data suggest a subtle, concerted role for SIRT2 and SIRT3 to be considered when developing drugs targeting multiple sirtuins.

Additive or synergistic effects between SIRT2 and SIRT3 could be anticipated since, for example, both SIRT2 and SIRT3 impact on ROS detoxification albeit through different transcriptional and post-transcriptional mechanisms, and both have been reported to dampen inflammatory responses (13, 23–31, 38, 39, 41–44). However, most of the effects observed here were rather slight. The most consistent effect of SIRT2/3 deficiency on immune cell distribution was on B cells that were increased slightly in the bone marrow, the spleen, the blood and more strongly in the peritoneum. Whether these variations impact on the development of plasma cells and humoral responses will be addressed in future studies. An impact on the levels of natural antibodies might be expected since B-1a cells are main producers of natural polyreactive IgM antibodies biased toward bacterial and self-antigens (70). Considering that B-1 B cells cooperate to protect from pneumococcal diseases through the generation of anti-streptococcal natural antibodies by B-1a cells and the generation of acquired anticapsule response by B-1b cells (78), it will be interesting to test whether SIRT2/3^−/−^ mice are resistant to *Streptococcus pneumoniae* pneumonia (79).

The bone marrow of SIRT3^−/−^ mice, but not SIRT2^−/−^ mice, contained more B cells. Up to now, the role of sirtuins in B cell biology has been mainly studied using B cell lymphoma giving rise to contrasting observations. Overexpression of SIRT1 and SIRT2 promoted the survival of chronic lymphocytic leukemia (CLL) B cells and correlated with poor outcome in patients with CLL (80, 81). On the contrary, SIRT3 acts as a tumor suppressor since reduced SIRT3 expression was associated with B cell proliferation and worsening of patients with B cell malignancies (82). However, sirtuins may have a differential impact on malignant cells and primary cells.

Additional leukocyte subsets were affected in SIRT2/3^−/−^ mice, however with small organ specific effects, for example a reduced proportion of alternative monocytes in the bone marrow and spleen, of PMNs in the blood and spleen and of DCs in the spleen. In general, these effects were observed in one of the two parental mouse lines (SIRT2^−/−^ or SIRT3^−/−^ mice), indicative of dominant effects of the knockouts. A more robust effect was observed on NK cells. The expression of sirtuins in NK cells has not been reported, but the fact that caloric restriction modulated NK cell phenotype suggested that sirtuins may impact on NK cell development or functions (83). NK cells from the blood of SIRT2^−/−^, SIRT3^−/−^, and SIRT2/3^−/−^ mice showed increased expression of the homing receptor CD62L, while the peritoneum of SIRT2/3^−/−^ mice contained less NK cells with reduced expression of CD43. CD43 is a maturation marker of NK cells, and reduced CD43 expression reflects decreased IFNγ production by NK cells (84). Thus, in the peritoneum, SIRT2/3^−/−^ NK cells potentially display a reduced inflammatory profile. It will be interesting to assess the functional consequences of SIRT2/3 deficiency on NK cell-mediated immunity, for instance in models of viral infections induced by murine cytomegalovirus (MCMV), lymphocytic choriomeningitis virus (LCMV) or influenza virus during which NK cells play a protective role. Whether SIRT2/3 deficiency affects NK cell antitumor cell cytotoxicity and has a role in graft-vs-host-disease would be additional interesting areas of investigations.

We previously reported that SIRT2^−/−^ and SIRT3^−/−^ BMDMs produced normal levels of a large panel of cytokines (13, 43). Conversely, SIRT2/3^−/−^ BMDMs produced increased levels of both proinflammatory (TNF, IL-6) and anti-inflammatory (IL-10) cytokines when compared to control BMDMs. A plausible mechanism underlying augmented cytokine response was the increased activation of ERK1/2 and NF-κB p65 intracellular signaling in SIRT2/3^−/−^ BMDMs. Like SIRT2^−/−^ BMDMs (13), SIRT2/3^−/−^ BMDMs phagocytosed better inert beads and live bacteria than control BMDMs. Moreover, they killed ingested *E. coli* to a higher rate. Of note, divergent observations have been reported in the literature, with SIRT2 and SIRT3 associated with both anti-inflammatory and proinflammatory activities also in macrophages (13, 23–31, 41–44). As an example of a possible antagonism between SIRT2 and SIRT3, silencing SIRT2 reduced NF-κB activation and induced macrophage alternative (anti-inflammatory) activation (85) while SIRT3 upregulation reduced macrophage inflammatory responses (86). This dichotomy might explain the mixed phenotype of SIRT2/3^−/−^ BMDMs which displayed characteristics of classically and alternatively activated macrophages.

Sirtuins are intrinsically linked to cell metabolism. The deletion of SIRT2 and SIRT3 stimulated HIF-1α expression and activity, thereby promoting Glut1 expression, glucose uptake and tumor growth (87, 88). In line, Hif1a mRNA levels were increased 1.6-fold in SIRT2/3^−/−^ BMDMs. However, this increase did not translate into an increase of glucose metabolism since Glut1 expression and glycolytic parameters, measured using the Seahorse technology, were all reduced in SIRT2/3^−/−^ BMDMs. This was surprising since inflammatory cytokine response is normally supported by glycolysis. Yet one has to take into account that SIRT2/3^−/−^ BMDMs were not purely “proinflammatory” since they increased IL-10 production, which in alternatively activated macrophages is fueled by oxidative phosphorylation (73). Nonetheless, glucose consumption and lactate production measured by luminescent assays were not affected in SIRT2/3^−/−^ BMDMs. Differences in the time point analyzed, plastic support and medium used for cell culture in relation with the technology used may have affected metabolic measurements. Overall, dual deletion of SIRT2 and SIRT3 may have disrupted metabolic control in BMDMs. A possible explanation for our observations is that post-translational mechanisms destabilized HIF-1α and impaired its transcriptional activity in SIRT2/3^−/−^ BMDMs. Attractive mediators could be SIRT6 and SIRT7 which expression was enhanced to some extent in SIRT2/3^−/−^ BMDMs. Indeed, SIRT6 functions as a co-repressor of HIF-1α, and SIRT7 inhibits the activity of HIF-1α through a mechanism independent of proteasomal or lysosomal degradation of HIF-1α (89, 90). SIRT2/3^−/−^ BMDMs were less dependent on FA and glutamine but more flexible toward FA, indicating that both glycolysis and FA metabolism were altered in SIRT2/3^−/−^ BMDMs. The expression of genes essential for the cholesterol synthesis pathway was decreased in SIRT2/3^−/−^ BMDMs, signifying that SIRT2/3 deficiency influenced lipid metabolism of macrophages by favoring FAO over cholesterol synthesis. Although counterintuitive at first glance, a similar antagonism between FAO and cholesterol synthesis has been observed in macrophages deficient in carnitine palmitoyltransferase 1 and 2 (CPT1, CPT2) (91).

Metabolic adaptation shapes immune cell functions and influences the acute phase and the resolution phase of inflammation that are fueled predominantly by glycolysis and oxidative phosphorylation, respectively (92, 93). Indeed, mice treated with 2-deoxyglucose to block glycolysis or with the sirtuin inhibitor cambinol were protected from endotoxemia (94, 95). Going well along with a reduced glycolytic activity of macrophages *in vitro*, SIRT2/3^−/−^ mice had a strong survival advantage during endotoxemia, which was associated with reduced blood levels of cytokines. Even so, SIRT2/3 double deletion did not seem to affect endotoxin tolerance as reported for SIRT1, SIRT4, and SIRT5 (24, 75–77). Interestingly, SIRT6, together with SIRT1, coordinated the switch from glucose to FAO during the acute inflammatory response (50), and transcriptional activation of SIRT6 via FOXO3a inhibited the Warburg effect in glioblastoma cells (96). Consequently, the increased expression of SIRT6 in SIRT2/3^−/−^ macrophages may favor FAO and contribute to dampen the cytokine storm involved in the pathological process of endotoxemia. An additional possibility is about a role played by B-1a cells, which were around 3-times more numerous in the peritoneum of SIRT2/3^−/−^ mice. Indeed, B-1a cells are an important source of IL-10 and may circumvent local cytokine burst in response to LPS challenge. Further, B-1a cells were shown to protect mice from experimental sepsis (97, 98). Finally, peritoneal NK cells in SIRT2/3^−/−^ mice were not only less, but also likely less activated as pinpointed by a reduced expression of CD43. As a consequence, the NK compartment potentially produced reduced levels of IFNγ which was shown to strongly potentiate TNF production and mortality during experimental endotoxemia (84, 99, 100). Altogether, several factors likely contributed to reduce systemic inflammatory parameters, morbidity and mortality of SIRT2/3^−/−^ endotoxemic mice.

Overall, we report that SIRT2/3 dual deletion revealed a phenotype not observed in single deficient mice, indicating that sirtuins act in concert or compensate each other for certain immune functions. Considering the link between SIRT2 and SIRT3, metabolism and age-associated dysfunctions, it will be of great interest to investigate the impact of SIRT2/3 deficiency in the pathogenesis of metabolic, oncologic, neurodegenerative and chronic inflammatory disorders. Importantly from a translational perspective, SIRT2/3^−/−^ mice were protected from endotoxemia. Thus, inhibitors targeting multiple sirtuins developed for clinical purposes may be useful to treat inflammatory diseases.

## Data Availability Statement

All datasets generated for this study are included in the article/Supplementary Material.

## Ethics Statement

The animal study was reviewed and approved by Service des Affaires Vétérinaires, Direction Générale de l'Agriculture, de la Viticulture et des Affaires Vétérinaires (DGAV), état de Vaud (Epalinges, Switzerland) under authorizations 876.9 and 877.9.

## Author Contributions

TH, EC, ER, JR, and DL performed the *in vitro* experiments. TH, EC, and DL performed the *in vivo* experiments. TH, EC, and TR conceived the project and designed the experiments. TH and TR wrote the paper. All the authors revised the paper.

### Conflict of Interest

The authors declare that the research was conducted in the absence of any commercial or financial relationships that could be construed as a potential conflict of interest.

## Supplementary Material

Supplementary Figure 1 Full blots used to create Figure 1.

Supplementary Figure 2 mRNA expression levels of phagocytic receptors by resting BMDMs. ItgaM, Itgb1, Itgb2, Itga5, and ItgaX mRNA levels were quantified by RT-qPCR and normalized to actin mRNA levels. Data are mean ± SD of four mice analyzed in triplicate.

Supplementary Figure 3 Gating strategies for flow cytometry analyses.
