# Supplementary material for: Dual Deletion of the Sirtuins SIRT2 and SIRT3 Impacts on Metabolism and Inflammatory Responses of Macrophages and Protects From Endotoxemia
Source: Front Immunol. 2019 Nov 26;10:2713. doi: 10.3389/fimmu.2019.02713 (PMC6901967; doi:10.3389/fimmu.2019.02713)

Supplementary Figure S1: Full blots used to create Figure 1B

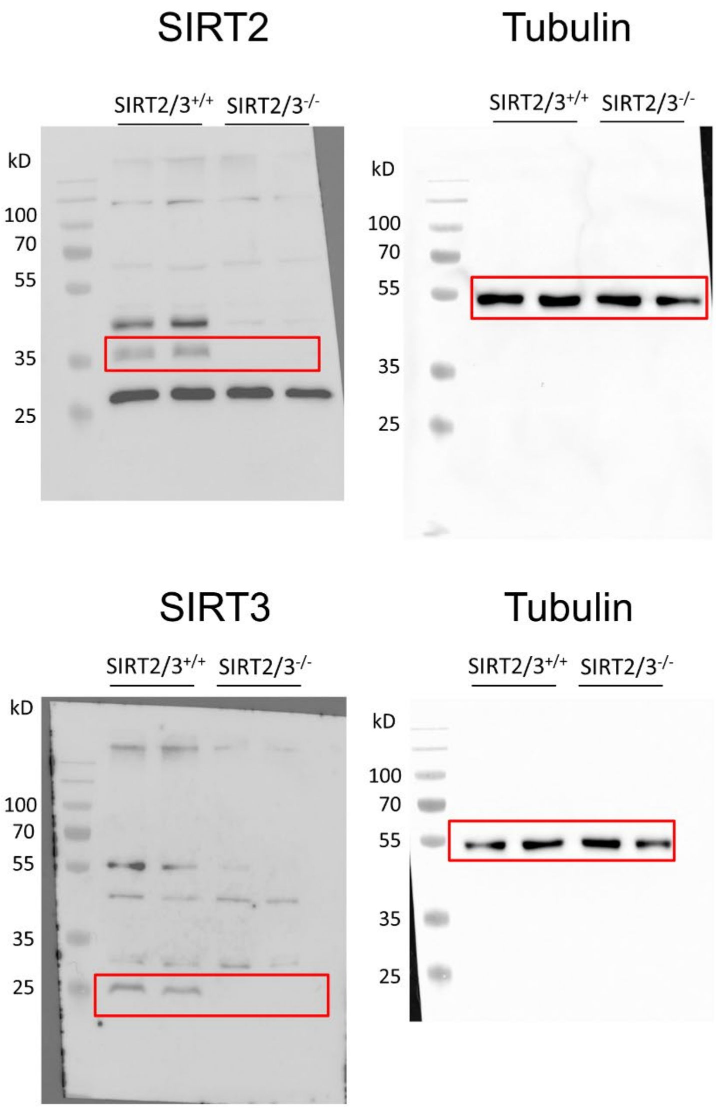

Supplementary Figure S1: Full blots used to create Figure 3B

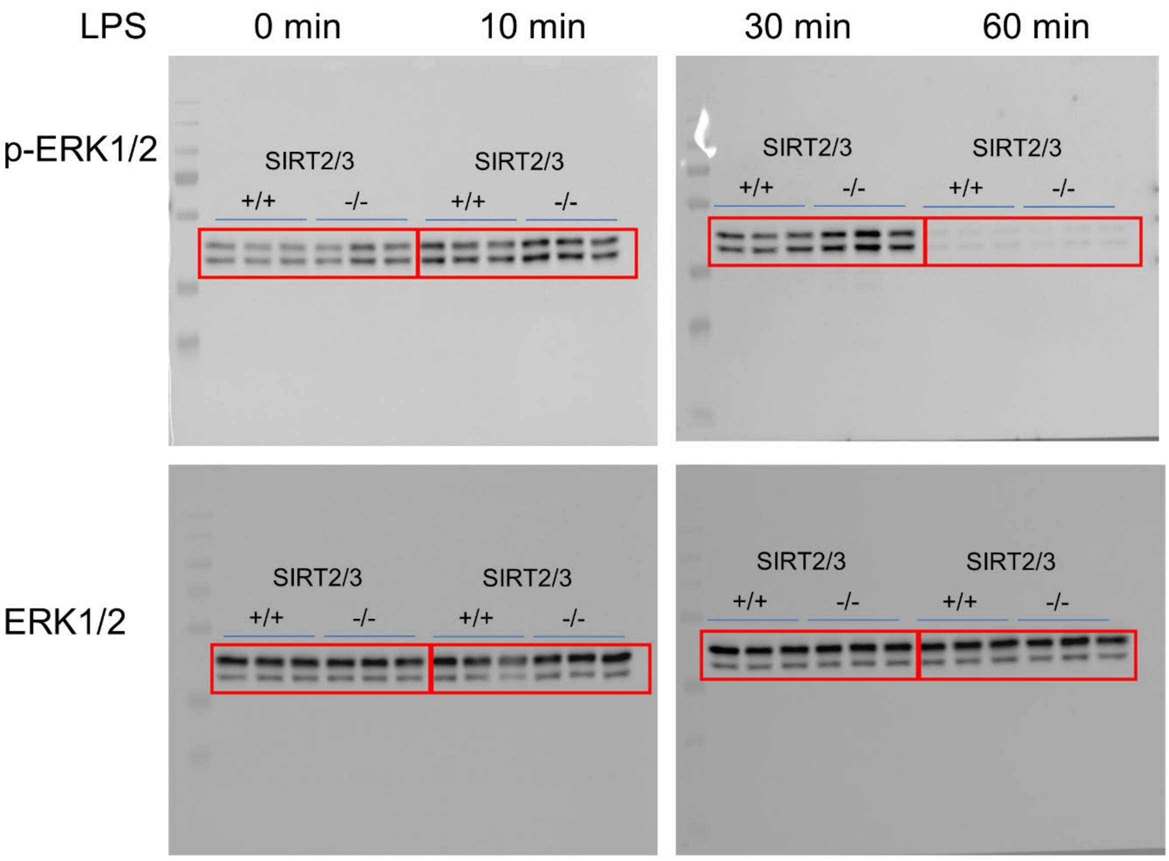

Supplement: Supplementary Figure 1 — Full blots used to create Figure 1. [file Image_1.pdf]
