# Supplementary figures and images for: Dual Deletion of the Sirtuins SIRT2 and SIRT3 Impacts on Metabolism and Inflammatory Responses of Macrophages and Protects From Endotoxemia
Source: Front Immunol. 2019 Nov 26;10:2713. doi: 10.3389/fimmu.2019.02713 (PMC6901967; doi:10.3389/fimmu.2019.02713)

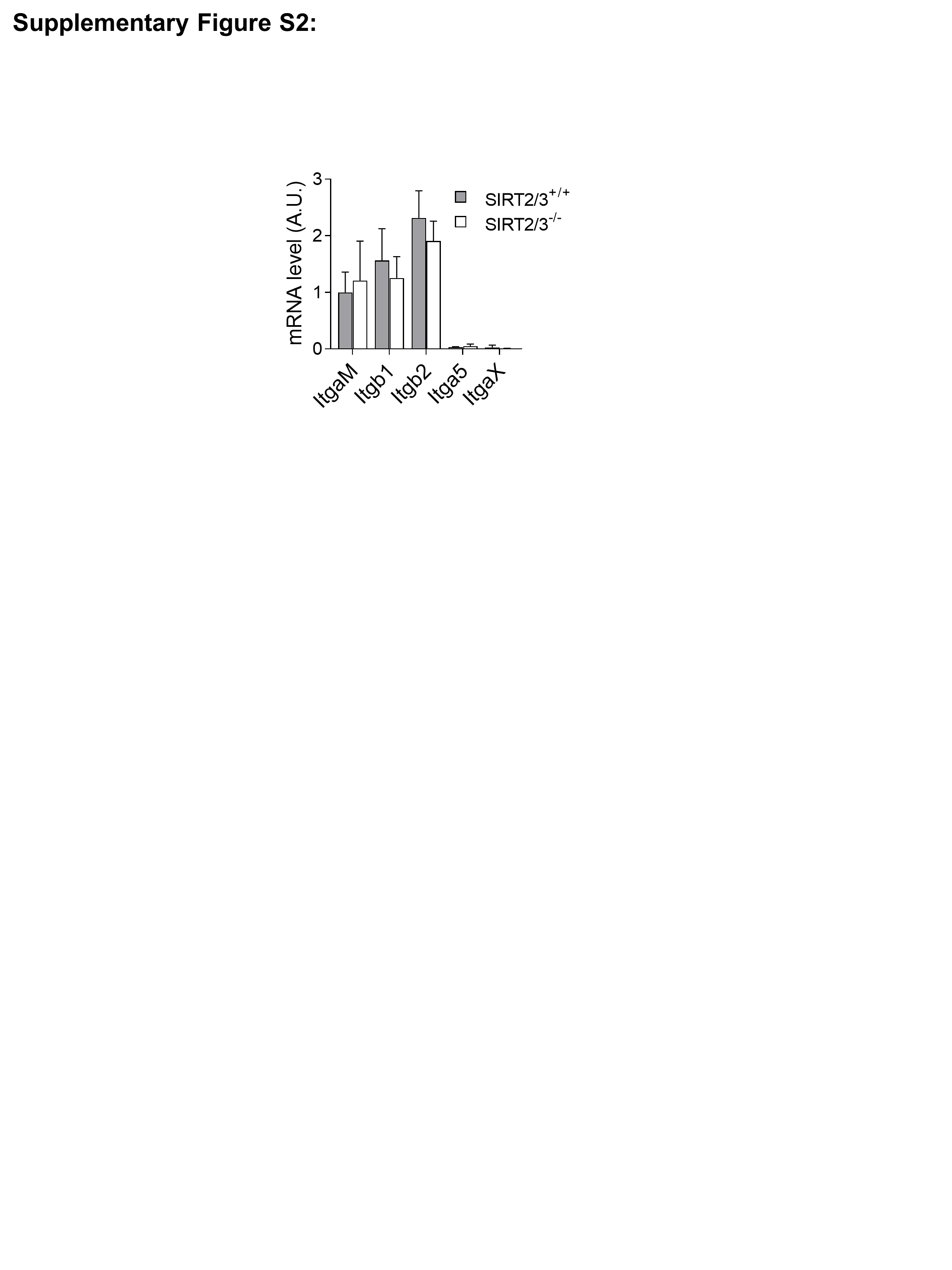

Supplement: Supplementary Figure 2 — mRNA expression levels of phagocytic receptors by resting BMDMs. ItgaM, Itgb1, Itgb2, Itga5, and ItgaX mRNA levels were quantified by RT-qPCR and normalized to actin mRNA levels. Data are mean ± SD of four mice analyzed in triplicate. [file Image_2.tif]

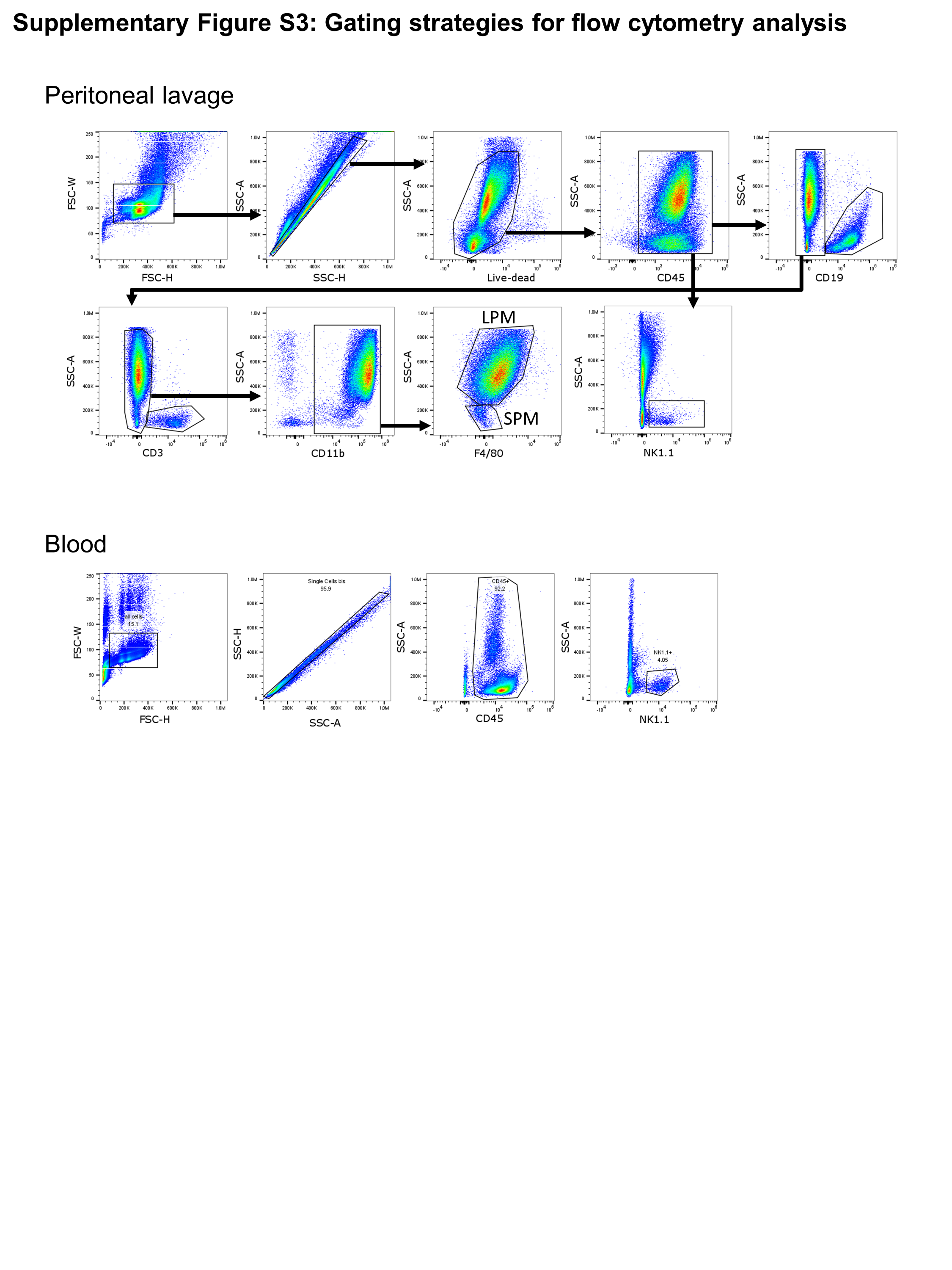

Supplement: Supplementary Figure 3 — Gating strategies for flow cytometry analyses. [file Image_3.tif]
