# Supplementary material for: Dual Deletion of the Sirtuins SIRT2 and SIRT3 Impacts on Metabolism and Inflammatory Responses of Macrophages and Protects From Endotoxemia
Source: Front Immunol. 2019 Nov 26;10:2713. doi: 10.3389/fimmu.2019.02713 (PMC6901967; doi:10.3389/fimmu.2019.02713)
Supplement: Supplementary file 4 [file Table_1.DOCX]

**Supplementary Information**

**1. Primers used for genotyping**

| **Genotype** | **Primer number** | **Sequence (5’-3’)** |
| --- | --- | --- |
| *Sirt2* | 831 | CTCCTTCAGTCATGAGGATTCCAGG |
| *Sirt2* | 949 | CTCTGGAAAAGGAGGAGGTGTTCTAC |
| *Sirt3* | 903 | GCTATGTAAGCACACAAGCTCACCTT |
| *Sirt3* | 947 | GATTCCTGATGCAAGATGGTTCTGTGC |
| *Sirt3* | 948 | CAGTCTTAGGCTAGCAAGAGTGAGG |

**2. Oligonucleotides used to quantify mRNA levels**

| **Target** | **Forward (5’-3’)** | **Reverse (3’-5’)** |
| --- | --- | --- |
| Arg1 | AAAGGAAAGTTCCCAGATGTACCA | TTTATTATATAGTGTTCCCCAGGGTCTAC |
| Cd206 | TGGTTGGGACTGACCTATGGA | TCACCGTAAGCCCAATTTTCAT |
| Cpt1 | CAAACCTATTCGTCTTCTGGGATCT | TGAAGAGTCGCTCCCACTGA |
| Fabp4 | TGGGAACCTGGAAGCTTGTC | GCAAAGCCCACTCCCACTT |
| Glut-1 | GTGGGCATGTGCTTCCAGTA | ACGAGGAGCACCGTGAAGAT |
| HIF-1a | GGCAGCGATGACACAGAAACTGA | TGATCTTGAATCTGGGGCATGGT |
| Hmgcr | CCAAACCCCGTAACCCAAA | CGACTATGAGCGTGAACAAGGA |
| ItgaM | CAACAAGCAGGTCTAGATGGT | GTGAGCCACACACAGAGCTTGCT |
| ItgaX | GGCTATCAAGCATGTCATAACAGAAC | CCCCTTGTTTTCTCCCATCA |
| Itgb1 | TGGAGAAAACTGTGATGCCGTAT | GCTGGTGCAGTTTTGTTCACTT |
| Itgb2 | CCACCGATGTGTGAGGATTG | CGGCACAAGAGGTGTGGTT |
| Itga5 | GAGGAGCCTGTGGAGTACAAGTC | GGGTCATTCTGTGGGTCCTTT |
| mTOR | CCAACCAGCCAATCATTCG | GCTTGGATGTGATGACTTGCA |
| Nos2 | GACAGCACAGAATGTTCCAGAATC | CCCCAGTTTTTGATCCTCACATA |
| Mvd | GACCAGCTAAAAACGACCACAAC | CCTCCTCGCGACCATTCA |
| Sirt1 | agggaacctttgcctcatct | gaggtgttggtggcaactct |
| Sirt4 | cgagcaaaagctcccaatag | gatcttgagcagcggaactc |
| Sirt5 | ggccgagtttaacatggaga | ccgttagtgccctgctttag |
| Sirt6 | acctgcaacccacaaaacat | ggctcagccttgagtgctac |
| Sirt7 | cacatgagcatcacccgttt | agcccatcacagttctgagaca |
| Sqle | GGAAGAGCCTCATCTCCAGTAAAG | CTGTGGTGCATCCTTCATAAGG |

**3. Antibodies and dyes used for flow cytometry**

| **Target** | **Clone** | **Coupling** | **Brand** | **Reference** |
| --- | --- | --- | --- | --- |
| B220 | RA-6B2 | Brilliant Violet 570 | BioLegend | 103237 |
| CD11b | M1/70 | APC-eFluor780 | eBioscience | 47-0112-82 |
| CD11b | M1/70 | PerCP-Cy5.5 | eBioscience | 45-0112-82 |
| CD11b | M1/70 | PE-Cy7 | BioLegend | 101216 |
| CD11c | N418 | APC | BioLegend | 117310 |
| CD11c | N418 | PE | BioLegend | 117307 |
| CD19 | 1D3 | PE-Cy7 | eBioscience | 25-0193-82 |
| CD23 | B3B4 | Alexa Fluor 700 | BioLegend | 101631 |
| CD25 | PC61 | PerCP-Cy5.5 | BD Biosciences | 551071 |
| CD3e | 17A2 | APC | eBioscience | 17-0032-82 |
| CD3e | 17A2 | PE-Cy7 | BioLegend | 100220 |
| CD4 | GK1.5 | APC-Cy7 | BioLegend | 100414 |
| CD43 | S7 | PE | BD Biosciences | 561857 |
| CD44 | IM7 | eFluor450 | eBioscience | 48-0441-82 |
| CD45 | 30-F11 | FITC | BD Biosciences | 553079 |
| CD5 | 53-7.3 | FITC | eBioscience | 11-0051-82 |
| CD62L | MEL-14 | PerCP-Cy5.5 | BD Biosciences | 560513 |
| CD8a | 53-6.7 | FITC | BioLegend | 100706 |
| IgD | 11-26c | PE | eBioscience | 12-5993-82 |
| Ly6C | HK1.4 | APC | BioLegend | 128016 |
| Ly6C | HK1.4 | PerCP-Cy5.5 | eBioscience | 45-5932-82 |
| Ly6G | 1A8 | eFluor450 | eBioscience | 48-9668-80 |
| Fixable Aqua |  |  | ThermoFisher | L34957 |
| Fixable Violet |  |  | ThermoFisher | L34955 |
| Fixable yellow |  |  | BioLegend | 77168 |
